# Myosin VI in the nucleolus of neurosecretory PC12 cells: its involvement in the maintenance of nucleolar structure and ribosome organization

**DOI:** 10.3389/fphys.2024.1368416

**Published:** 2024-05-07

**Authors:** Jolanta Nowak, Robert Lenartowski, Katarzyna Kalita, Lilya Lehka, Olena Karatsai, Marta Lenartowska, Maria Jolanta Rędowicz

**Affiliations:** ^1^ Laboratory of Molecular Basis of Cell Motility, Nencki Institute of Experimental Biology, Polish Academy of Sciences, Warsaw, Poland; ^2^ Faculty of Biological and Veterinary Sciences, Nicolaus Copernicus University in Torun, Torun, Poland; ^3^ Centre for Modern Interdisciplinary Technologies, Nicolaus Copernicus University in Torun, Torun, Poland; ^4^ Laboratory of Neurobiology, Nencki-EMBL Partnership for Neural Plasticity and Brain Disorders—BRAINCITY, Nencki Institute of Experimental Biology, Polish Academy of Sciences, Warsaw, Poland

**Keywords:** actinomycin D, B23, fibrillarin, myosin VI, nucleolin, nucleolus, nucleolar stress, PC12 cells

## Abstract

We have previously shown that unconventional myosin VI (MVI), a unique actin-based motor protein, shuttles between the cytoplasm and nucleus in neurosecretory PC12 cells in a stimulation-dependent manner and interacts with numerous proteins involved in nuclear processes. Among the identified potential MVI partners was nucleolin, a major nucleolar protein implicated in rRNA processing and ribosome assembly. Several other nucleolar proteins such as fibrillarin, UBF (upstream binding factor), and B23 (also termed nucleophosmin) have been shown to interact with MVI. A bioinformatics tool predicted the presence of the nucleolar localization signal (NoLS) within the MVI globular tail domain, and immunostaining confirmed the presence of MVI within the nucleolus. Depletion of MVI, previously shown to impair PC12 cell proliferation and motility, caused disorganization of the nucleolus and rough endoplasmic reticulum (rER). However, lack of MVI does not affect nucleolar transcription. In light of these data, we propose that MVI is important for nucleolar and ribosome maintenance but not for RNA polymerase 1-related transcription.

## 1 Introduction

Myosins are actin-based ATP-dependent molecular motors involved in a panoply of cellular processes associated with motile and contractile processes. They are classified into over 30 families (classes) based on differences in a primary sequence of the ATP- and actin-binding motor domain, engaged in force generation (Odronitz and Kollmar, 2007). The best characterized and most abundant of myosins are muscle myosins, which together with the so-called non-muscle isoforms (resembling classical muscle counterparts) form class II, also termed as conventional myosins. All other myosins, very diverse in their structure and function, are termed as unconventional ones. Myosins are mainly known to function in the cytoplasm; however, it has been shown that several unconventional myosins are present in the nucleus, where they play important roles in numerous nuclear processes (de Lanerolle, 2012; Belin and Mullins, 2013; Sarshad and Percipalle, 2014). Among them are nuclear myosin IC (NMIC, isoforms b and c), myosins VA and VB, myosin VI, myosin XVIB, and myosins XVIIIA and XVIIIB. In the nucleus, these myosins are believed to interact with nuclear actin and participate in intra-nuclear trafficking, DNA replication and repair, as well as transcription (Shahid-Fuente and Toseland, 2023; Cook and Toseland, 2021; Cook et al., 2020; Caridi et al., 2018). It is noteworthy that three isoforms, nuclear myosin IC (NMI), myosin VA, and myosin VB, have been found in the nucleolus (Lindsay and McCaffrey, 2009; Philimonenko et al., 2004; Pranchevicius et al., 2008). However, molecular mechanisms of involvement of these myosins in nucleolar processes still remain poorly understood.

**FIGURE 1:**
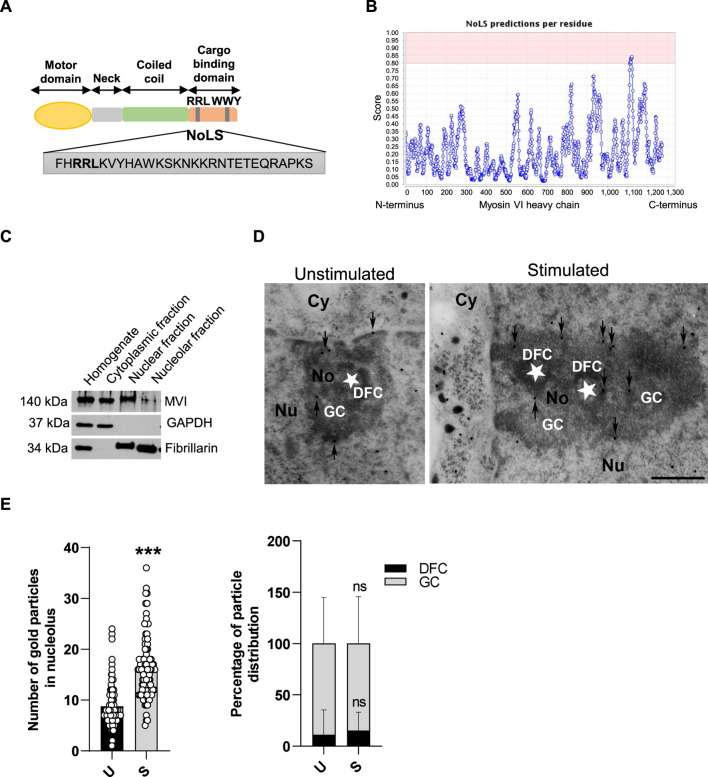
MVI is present within the nucleolus of PC12 cells. **(A)** Schematic diagram of the organization of the MVI heavy chain with depiction of the putative NoLS (nucleolar localization sequence) predicted by the NoD detector; detailed explanation is provided in the text. **(B)** Schematic diagram presenting NoD analysis with localization of a putative NoLS (a region with a score > 0.8; shown in pink) within the MVI heavy chain globular tail domain. The numbers on the *x*-axis correspond to amino acid positions of the MVI heavy chains. **(C)** Subcellular fractionation of PC12 cells. MVI is detectable in the nucleolar fraction. GAPDH, cytoplasmic protein marker, was detected only in the cytoplasmic fraction, but not in the nuclear and nucleolar fractions. Fibrillarin, nucleolar protein marker, was detected in both in the nuclear and nucleolar fractions. **(D)** Assessment of the sub-nucleolar distribution of MVI by means of immunogold electron microscopy in unstimulated and stimulated cells. Labeling was concentrated in the dense fibrillar component (DFC) surrounding the fibrillar center (asterisk) and in the granular component (GC) of the nucleolus. Cy, cytoplasm; Nu, nucleus; No, nucleolus. Bars, 1 µm. **(E)** Quantification of the gold particles in the nucleoli of PC12 cells before (U) and after stimulation with 56 mM KCl (S); ***, *p* < 0.001 (left graph). Quantification of distribution of the gold particles within DFC and GC subdomains (right graph). ns, not statistically significant. 100% corresponds to all the particles spotted in the nucleoli at given conditions. Quantitative analyses were based on ∼100 images of nucleoli of unstimulated and stimulated cells from three different cell cultures. For quantification of distribution of MVI in nucleolar subdomains, the analyses were carried out for 20 nucleoli of two experimental conditions.

Myosin VI (MVI), present in the nucleus, is the only known myosin moving toward the minus (pointed) end of actin filaments (Wells et al., 1999; Sweeney and Houdusse, 2010). Similar to other myosins, MVI heavy chain (MW ∼140 kDa) contains the N-terminal motor domain, a neck region, and the C-terminal tail domain involved in cargo binding (Avraham et al., 1995; de Jonge et al., 2019; Li et al., 2016) (Figure 1A).

In the cytoplasm, it acts as a transporting motor or an anchor linking vesicles and/or plasma membrane proteins to the actin cytoskeleton (Sweeney and Houdusse, 2007; 2010; Chibalina et al., 2009; Li et al., 2016). It plays important roles in endocytosis, cell motility, and adhesion, as well as in the maintenance of membranous compartments such as the Golgi apparatus and endoplasmic reticulum (ER) (Chibalina et al., 2009; Warner et al., 2003; Karolczak et al., 2015; Zakrzewski et al., 2021). It has been shown that in mice and humans, loss or point mutations within the MVI gene (*MYO6*) lead to deafness as well as mild defects in several organs, including the brain, heart, kidney, intestines, testis, and skeletal muscles (Avraham et al., 1997; Osterweil et al., 2005; Mohhidin et al., 2004; Hegan et al., 2015; Karatsai et al., 2023; Gotoh et al., 2010; Ameen and Apodaca 2007; Zakrzewski et al., 2021; Lehka et al., 2022). In addition, a significant increase in its synthesis was detected in highly malignant cancers, suggesting its important role in cell proliferation (Yoshida et al., 2004; Dunn et al., 2006; Zhan et al., 2023).

In the nuclei of numerous cancer cell lines, MVI was found to localize to chromatin-free regions, where it was associated with the RNA polymerase II (Pol2) transcription machinery (Jung et al., 2006; Vreugde et al., 2006; Majewski et al., 2018). It was shown that in the nucleus of HeLa cells, MVI acts as the molecular anchor that holds Pol2 in high-density clusters, and perturbation of MVI leads to the disruption of Pol2 localization and chromatin organization (Hari-Gupta et al., 2022). These changes subsequently lead to a decrease in gene expression, suggesting that MVI plays a crucial role in the spatial regulation of gene expression (Hari-Gupta et al., 2022). Moreover, the same group showed that a direct binding of MVI to DNA is important for its interaction with Pol2 (Fili et al., 2017). In addition, several other reports demonstrated the involvement of MVI not only in gene transcription but also in gene pairing (Cho and Chen 2010; Loikkanen et al., 2009; Zorca et al., 2015).

In line with the aforementioned observations, our previous data demonstrate that in neurosecretory PC12 cells, MVI translocates, in a stimulation-dependent manner, to the nucleus, where it localizes to numerous nuclear compartments, including the nucleolus. It also interacts with a variety of proteins involved in nuclear (and nucleolar) functions, including nucleolin and ribosomal protein S6 (Majewski et al., 2018). In the present study, we addressed for the first time the functional significance of the presence of MVI within the nucleolus. Our data demonstrate that besides nucleolin, MVI interacts with several nucleolar proteins involved in rRNA synthesis and processing, including UBF (upstream binding factor), fibrillarin, and B23 (also termed nucleophosmin, NPM1). We show that MVI is involved in the maintenance of nucleolar integrity and ribosome localization at the ER membranes. However, contrary to NMIC (Philimonenko et al., 2004), MVI does not seem to be involved in pre-rRNA synthesis.

## 2 Materials and methods

### 2.1 Plasmids

Plasmids for the expression of the recombinant globular tail domain of MVI fused with GST (glutathione S-transferase) in *E. coli* was constructed by subcloning a fragment of the rat MVI nucleotide sequence (Majewski et al., 2012) (gene ID D4A5I9) corresponding to the MVI globular tail (aa 1046-1285) into the pGEX-4T1 vector (from GE Healthcare, Cat. No 28-9545-49). Glutathione Sepharose 4B was also obtained from GE Healthcare (Cat. No 17-0756-01).

### 2.2 Antibodies and fluorescent markers

The antibodies were used as follows: rabbit polyclonal antibody to MVI (Proteus, Cat. No 25-6791), mouse monoclonal antibody to β-actin (Sigma-Aldrich, Cat. No A3854), mouse monoclonal antibody to B23 (Abcam, Cat. No ab 10530), rabbit polyclonal antibody to GRP78 (Abcam, Cat. No 21685), mouse monoclonal antibody to fibrillarin (Thermo Fisher Scientific, Cat. No MA3-16771), mouse monoclonal antibody to glyceraldehyde-3-phosphate dehydrogenase (GAPDH, Millipore, Cat. No MAB 274), mouse monoclonal antibody to RPA 194 (Pol1) (Santa Cruz Biotechnology, Cat. No sc-48385), mouse monoclonal antibody to UBF (Santa Cruz Biotechnology, Cat. No sc-13125), goat polyclonal antibody to lamin B (Santa Cruz Biotechnology, Cat. No sc-6217), mouse monoclonal antibody to p-S6 (Cell Signaling, Cat. No 62016), mouse monoclonal antibody to S6 (Cell Signaling, Cat. No 2317), rabbit monoclonal antibody to p-p70S6K (Cell Signaling, Cat. No 9234), rabbit monoclonal antibody to p70S6K (Cell Signaling, Cat. No 2708), goat anti-mouse IgG antibody, HRP conjugate (Millipore, Cat. No AP308P), goat anti-rabbit IgG antibody, HRP conjugate (Millipore, Cat. No AP307P), and donkey anti-goat IgG antibody, HRP conjugate (Santa Cruz Biotechnology, Cat. No sc-2020).

VECTASHIELD PLUS Antifade Mounting Medium with DAPI was obtained from Vector Laboratories (Cat. No H2000). For immunofluorescence studies, the following secondary antibodies were used: goat anti-rabbit IgG labeled with Alexa Fluor 488 (Invitrogen, Cat. No A11008) and goat anti-mouse IgG labeled with Alexa Fluor 546 (Invitrogen, Cat. No A11003).

The *in situ* proximity ligation assay (PLA) kit was purchased from Sigma-Aldrich (kit components: Duolink In Situ PLA Probe Anti-Mouse MINUS, Cat. No DUO 92004; Duolink In Situ PLA Probe Anti-Rabbit PLUS, Cat. No DUO 92002; Duolink In Situ Detection Reagents Red, Cat. No DUO 92008; Duolink In Situ Wash Buffers, Cat. No DUO 82049; and Duolink In Situ Mounting Medium with DAPI, Cat. No DUO 92006).

### 2.3 Cell culture

The non-adherent variant of PC12 cells (American Type Cell Culture Collection, ATCC, Cat. No CRL-1721) was cultured in RPMI media (Gibco, Cat. No 52400025) containing 2 mM L-glutamine, 4.5 g/L glucose supplemented with 10% heat-inactivated horse serum (Gibco, Cat. No 26050088), 5% heat-inactivated fetal bovine serum (Gibco, Cat. No 10270106), and antibiotics: 1% penicillin/streptomycin (Gibco, Cat. No 15140-122) at 37°C in humidified air containing 5% CO_2_.

In addition, stable MVI knockdown (MVI-KD) and a control scrambled cell lines (control), both obtained earlier by Dr. Ł. Majewski (Majewski et al., 2011), were used. These lines were prepared using a plasmid encoding *sh*RNA directed against the MVI mRNA and a plasmid encoding a control *sh*RNA not recognizing any known mammalian mRNA sequences (Majewski et al., 2011). MVI-KD and control PC12 cells were cultured in F12K (Kaighn’s Modification of Ham’s F-12) (ATCC, Cat. No 30-2004) containing 2 mM L-glutamine, 1.5 g/L sodium bicarbonate supplemented with 2.5% heat-inactivated fetal bovine serum (Gibco, Cat. No 10270106), 15% heat-inactivated horse serum (Gibco, Cat. No 26050088), antibiotics: 1% penicillin/streptomycin (Gibco, Cat. No 15140-122), and hygromycin B as a selective antibiotic (250 ng/mL) at 37°C in humidified air containing 5% CO_2_.

Cells were lysed in an ice-cold buffer that contained 50 mM Tris–HCl pH 7.5 (Sigma-Aldrich, Cat. No T6687), 150 mM NaCl (Chempure, Cat. No 117941206), 0.1% Triton X-100 (Sigma-Aldrich, Cat. No SLCJ7494), 2 mM EGTA (Sigma-Aldrich, Cat. No E3889), 1 mM DTT (Sigma-Aldrich, Cat. No 10197777001), 1 mM PMSF (Sigma-Aldrich, Cat. No P7626), cOmplete™ Protease Inhibitor Cocktail (Roche, Cat. No 04693132001), and phosphatase inhibitor PhosSTOP™ (Roche, Cat. No 4906845001).

### 2.4 Subcellular fractionation

To obtain the cytoplasmic, nuclear, and nucleolar fractions, PC12 cells were subjected to fractionation according to the Hacot protocol (Hacot et al., 2010) with several modifications. Briefly, cells were washed with PBS, harvested, and centrifuged at 200 *g* for 3 min at RT. The pellet was resuspended in a hypotonic buffer consisting of 10 mM HEPES, pH 7.9, 10 mM KCl, 1.5 mM MgCl_2_, and 0.5 mM DTT and kept on ice for 15 min to induce osmotic shock, causing cell membrane disruption. The suspension was homogenized using a Dounce-type glass tissue homogenizer and centrifuged at 1,200 *g* for 5 min at 4°C. The resultant supernatant contained the cytoplasmic protein fraction, while the pellet contained both cell debris and cell nuclei. The pellet was then subjected to centrifugation in the sucrose gradient: it was resuspended in S1 buffer (0.25 M sucrose and 10 mM MgCl_2_), and the suspension was placed in centrifuge tubes containing S2 buffer (0.88 M sucrose and 0.5 mM MgCl_2_) and centrifuged at 1,200 *g* for 5 min at 4°C. The supernatant was discarded, and the pellet at the bottom of the tube (representing the purified fraction of cell nuclei) was resuspended in buffer S3 (0.35 M sucrose and 0.5 mM MgCl_2_). Next, the nucleolus fraction was obtained by sonication of the purified nuclei fraction using a S-250D sonicator (Branson Ultrasonic S.A.) with a 1/8'' (3.2 mm) microtip at 30% power of the device in three cycles/sequences (10-s sonication and 10-s pause). The suspension that resulted upon sonication was applied to the surface of the S2 buffer and centrifuged at 2,000 *g* for 20 min at 4°C. The supernatant contained the nucleoplasmic fraction, and the pellet contained the nucleolar fraction, which was suspended in S3 buffer. All the obtained fractions were subjected to SDS-PAGE, followed by immunoblotting analysis for the presence of MVI and other marker proteins (GAPDH for the cytoplasm, and fibrillarin for the nucleoplasm and nucleolus) used as the internal loading control and indicators of fraction purity. The protein concentration was determined using the standard Bradford method.

### 2.5 Cell stimulation

To induce secretion, PC12 cells were cultured as described above and stimulated essentially according to Vitale et al. (1992) and Trifaró and Lee (1980). Treatment with 56 mM KCl is generally accepted as a method for *in vitro* PC12 cell stimulation as high concentrations of external KCl cause PC12 cell plasma membrane depolarization and evoke catecholamine release. Briefly, cells were washed with Locke’s solution containing 2.6 mM KCl, 154 mM NaCl, 2.2 mM CaCl_2_, 0.5 mM KH_2_PO_4_, 1.25 mM K_2_HPO_4_, 1.2 mM MgCl_2_, and 10 mM glucose. Then, they were incubated in Locke’s solution with elevated K^+^ concentration (56 mM KCl, 103.6 mM NaCl, 2.2 mM CaCl_2_, 0.5 mM KH_2_PO_4_, 1.25 mM K_2_HPO_4_, 1.2 mM MgCl_2_, and 10 mM glucose) to stimulate secretion or in calcium-free Locke’s solution (2.6 mM KCl, 154 mM NaCl, 0.5 mM KH_2_PO_4_, 1.25 mM K_2_HPO_4_, 1.2 mM MgCl_2_, and 10 mM glucose) to block the secretion. Cells were further processed for post-embedding immunogold MVI localization.

### 2.6 Actinomycin D treatment

To induce nucleolar stress, PC12 cells were treated with the Pol1 transcription inhibitor, actinomycin D (ActD) (Sigma-Aldrich, Cat. No A1410). Briefly, examined cells were incubated at 37°C for 3 h in the culture medium in the presence or absence of 0.05 μg/mL ActD and subjected to further analyses.

### 2.7 Immunoblot analysis

PC12 cell lysates and subcellular fractions were separated using 10% polyacrylamide SDS gels and then transferred to a nitrocellulose membrane (Bio-Rad, Cat. No 1620115). After the transfer, the membrane was blocked for 1 h at room temperature in TBS containing 5% non-fat milk powder or 5% BSA (Sigma-Aldrich, Cat. No A7906-150G) and 0.2% Triton X-100 followed by overnight incubation with appropriate dilutions (from 1:100 to 1:5,000) of different primary antibodies. The primary antibodies were detected using 1:10,000 dilutions of anti-rabbit (Millipore, Cat. No AP307P), anti-mouse (Millipore, Cat. No AP308P), or anti-donkey (Cat. No sc-2020, Santa Cruz Biotechnology) secondary antibodies conjugated with horse radish peroxidase. The reaction was developed using the ECL detection kit (Pierce, Cat. No 34095 and Millipore, Cat. No P90720). Usually, 10–20 μg of protein was loaded onto the gel. Band densitometry quantification was performed using the Fiji distribution of ImageJ 1.52a software (National Institutes of Health and the University of Wisconsin, Madison, WI, United States).

### 2.8 Immunolocalization studies

The distribution of MVI and other examined proteins in PC12 cells was evaluated by indirect immunocytochemistry. Cells on coverslips were fixed in 4% paraformaldehyde for 15 min, washed three times with phosphate-buffered saline (PBS) for 5 min, and blocked in a solution that contained 2% horse serum and 0.02% Triton X-100 in PBS for 1 h at room temperature. Coverslips were then incubated overnight at 4°C with rabbit polyclonal antibody to MVI, mouse monoclonal antibody to B23, rabbit polyclonal antibody to GRP78, mouse monoclonal antibody to fibrillarin, mouse monoclonal antibody to RPA 194 (Pol1), mouse monoclonal antibody to UBF, or mouse monoclonal antibody to S6 in a blocking solution and washed three times in PBS with 0.02% Triton X-100. This was followed by incubation with Alexa Fluor 488-conjugated anti-rabbit secondary antibody or Alexa Fluor 546-conjugated secondary anti-mouse antibody in a blocking solution for 60 min.

Finally, cells were washed three times in PBS with 0.02% Triton X-100 and mounted using VECTASHIELD PLUS Antifade Mounting Medium with DAPI. The specimens were visualized using a Zeiss LSM780 spectral confocal microscope equipped with a Plan-Apochromat 63x/1.40 Oil DIC M27 lens. In double immunostaining, special care was taken to control for any possible cross-reactivity (cross-bleeding) of the detection systems. We carefully adjusted the spectral ranges of detectors and always scanned the images sequentially. For negative controls, the primary antibody was omitted.

### 2.9 Confocal endoplasmic reticulum visualization

ER was visualized by staining with the ER-specific dye, ER Tracker™ Blue/White DPX (Thermo Fisher Scientific, Cat. No E12353), which is retained within the ER lumen, thus labeling the ER tubular network, according to the manufacturer’s instructions. Briefly, cells were seeded on glass coverslips and cultured for 24 h and then incubated for 30 min at 37°C and 5% CO_2_ with 1 μM ER tracker diluted in the culture medium. Then, the stained cells were fixed with 4% formaldehyde for 10 min, washed in PBS, and mounted using VECTASHIELD PLUS Antifade Mounting Medium without DAPI. Images were collected with the Zeiss LSM780, inverted Axio Observer Z.1 equipped with the 63x/1.4 Oil Plan-Apochromat DIC objective. A diode laser of 405 nm was used to excite fluorescence. Optical sections (2048 pixels × 2048 pixels × 8 Bit/pixel) were collected. The images were processed using ZEN Blue 2.1 software.

### 2.10 Ultrastructure of PC12 cells—transmission electron microscopy

PC12 cells were cultured on Thermanox™ coverslips (Electron Microscopy Sciences, Cat. No 72274) coated with poly-L-lysine (PLL, Electron Microscopy Sciences, Cat. No 19320) in RPMI-1640 medium supplemented with 10% HS, 5% FBS, and antibiotics: 1% penicillin/streptomycin or F12K medium supplemented with 2.5% FBS, 15% HS, and antibiotics: 1% penicillin/streptomycin, depending on the cell type. Cells were washed three times for 30 s each in PBS and fixed with 2% glutaraldehyde (GA) solution in PBS for 1 h at room temperature. Next, the fixed cells were washed three times for 10 min each in PBS, followed by post-fixation in 1% osmium tetroxide (OsO_4_) for 30 min at room temperature (OsO_4_ not only fixes but also provides contrast to lipid membranes). Sections were then rinsed twice for 10 min in PBS and twice for 5 min in deionized water, followed by dehydration in ethanol solutions of increasing concentrations in a so-called dehydration series, starting with 50% alcohol, followed by 70%, 80%, 90%, 96%, (5 min each) and anhydrous (99.8% absolute), twice for 15 min each, and then embedded in Spurr resin (Sigma-Aldrich, Cat. No EM0300) according to the standard protocol. The resin-submerged sections were cut into ultra-thin sections (60–70 nm thick) by using a diamond knife (Micro Star Technologies) and a Leica UTC ultramicrotome and collected on copper microscope grids (Electron Microscopy Sciences, Cat. No EMS400CU). The sections were stained with 2.5% uranyl acetate and 0.4% lead citrate and then examined by using a Joel EM 100 transmission electron microscope.

### 2.11 Post-embedding immunogold MVI localization

PC12 cells were grown on Thermanox™ coverslips, as described earlier. The cells were gently rinsed with PBS and fixed with 4% (v/v) formaldehyde and 0.25% (v/v) GA in the same PBS buffer for 1 h at room temperature. Fixed cells were washed three times with PBS, dehydrated in graded ethanol concentrations, and embedded in LR White resin (Electron Microscopy Sciences, Cat. No 14380) according to the standard protocol. Ultrathin sections were cut by using a diamond knife (Micro Star Technologies) and a Leica UTC ultramicrotome and collected on Formvar film-coated nickel grids (Electron Microscopy Sciences, Cat. No FCF400-Ni). The sections were then pretreated with 50 mM glycine in PBS for 10 min and incubated with a blocking solution containing 3% (w/v) bovine serum albumin (BSA) in PBS for 5 min at room temperature. Next, sections were placed in 1:50 dilution of a primary MVI antibody in PBS supplemented with 0.3% BSA for 2 h, followed by incubation with a gold-conjugated anti-rabbit IgG 15-nm secondary antibody (BB International, Cat. No R14003) at 1:100 dilution in PBS with 0.1% BSA for 30 min. Both incubations were carried out at room temperature. For the negative control, the primary antibody was omitted. Finally, the sections were stained with 2.5% uranyl acetate and examined on a JEOL JEM 1010 transmission electron microscope.

### 2.12 GST pull-down assay

The fusion proteins composed of GST and MVI C-terminal globular tail domain (GST-MVI-GD) as well as GST alone were purified, as described by Majewski et al. (2012). For the lysate, cells were lysed in an ice-cold buffer that contained 50 mM Tris (pH 7.5), 150 mM NaCl, 0.1% Triton X-100, 1 mM DTT, 2 mM EGTA, 50 mM NaF, 1 mM Na_3_VO_4_, and 1 mM PMSF and supplemented with the cOmplete™ Protease Inhibitor Cocktail and phosphatase inhibitor PhosSTOP™. The assay was performed as described by Majewski et al. (2012). Briefly, the lysates were precleared with GST-bound Glutathione Sepharose 4B beads for 2 h at 4°C to remove proteins non-specifically binding to Glutathione Sepharose 4B and/or GST and subsequently incubated with Glutathione Sepharose 4B beads bound to GST-MVI-GD or GST alone for 4 h at 4°C. The beads were exhaustively washed in the ice-cold lysate buffer, described above, and subjected to SDS–PAGE electrophoresis followed by immunoblotting.

### 2.13 Proximity ligation assay (PLA)

PC12 cells after fixation were blocked in the Duolink blocking solution in a humidity chamber for 30 min at 37°C and incubated with the following primary antibodies: rabbit polyclonal anti-MVI and mouse monoclonal antibodies anti-B23, anti-fibrillarin, anti-UBF, anti-Pol1, anti-p-S6, and anti-S6, diluted in Duolink Antibody diluent solution for 3 h at 37°C. Cells were next washed two times in a wash buffer for 5 min at room temperature. Next, secondary antibodies conjugated with oligonucleotides, PLA probe anti-mouse MINUS, and PLA probe anti-rabbit PLUS were applied in the Duolink antibody diluent solution for 1 h at 37°C and then washed twice for 5 min. The Duolink assay was further performed strictly according to the manufacturer’s instructions. For negative controls, the primary antibodies were omitted.

### 2.14 Co-immunoprecipitation

To perform co-immunoprecipitation, PC12 cells (CRL-1721) were lysed in a buffer containing 50 mM Tris (pH 7.5), 150 mM NaCl, 2 mM EGTA, 0.1% Triton X-100, 2 mM MgCl_2_, 2 mM MgATP, 50 mM NaF, and 1 mM Na_3_VO_4,_ supplemented with the cOmplete™ Protease Inhibitor Cocktail and phosphatase inhibitor PhosSTOP™. The lysates were pre-cleared with A/G agarose beads (Santa Cruz Biotechnology, Cat. No sc-2003) for 30 min at 4°C and subsequently incubated for 4 h at 4°C with 10 µg of the anti-MVI antibody (Proteus, Cat. No 25-6791) or non-immunized rabbit IgG (Santa Cruz Biotechnology, Cat. No sc-2027) as a control, followed by overnight incubation with the aforementioned agarose beads. Next, the beads were washed with the lysis buffer and then subjected to SDS–PAGE electrophoresis followed by immunoblotting with antibodies of interest to detect the co-immunoprecipitated complexes.

### 2.15 Quantitative real-time polymerase chain reaction (qRT-PCR)

RNA was isolated from 5 × 10^6^ PC12 cells (scrambled and MVI-KD) using the RNeasy Plus Universal Mini Kit (Qiagen, Cat. No 73404) according to the manufacturer’s instructions. DNA contamination from RNA samples was removed through treatment with RNase-Free DNase I (Qiagen, Cat. No 79254). First-strand cDNA synthesis was performed using 1 µg of RNA and the SuperScript™ III Reverse Transcriptase Kit (Thermo Fisher Scientific, Cat. No 18080-093) with random hexamers. Quantitative PCR was performed using the Fast SYBR Green Master Mix (Applied Biosystems, Cat. No 4385612) with an Applied Biosystems 7900HT Fast Real-Time PCR System. The oligonucleotide primer sequence used for rRNA analysis included 45S pre-rRNA—forward: 5′-TGG​GGC​AGC​TTT​ATG​ACA​AC-3´; 45S pre-rRNA—reverse: 5′-TAG​CAC​CAA​ACG​GGA​AAA​CC-3´;

18S rRNA—forward: 5′GTT​GGT​TTT​CGG​AAC​TGA​GGC3’;

18S rRNA—reverse: 5′GTC​GGC​ATC​GTT​TAT​GGT​CG3’.

Pre-rRNA and 18S rRNA levels were quantified using the ΔΔ CT method (2^−ΔΔCT^). Expression values were obtained from four independent experiments run in triplicates of each cDNA sample: 45S pre-rRNA relative to 18S rRNA (from the same cDNA preparations) (Kalita et al., 2008).

### 2.16 Identification of nucleolar localization signals in MVI

To identify a nucleolar localization signal (NoLS) within the MVI heavy chain, the NoD webserver was used (http://www.compbio.dundee.ac.uk/nod) (Scott et al., 2011).

### 2.17 Statistical analyses

All experiments were performed at least three times in two–three technical replicates. The results were expressed as means ± SD (standard deviation). If the data were normally distributed, we performed parametric two-tailed Student’s t-test or one-way ANOVA using GraphPad Prism 8.4.3 software (San Diego, CA, United States). Data that were non-normally distributed were analyzed with a nonparametric Mann–Whitney U-test to determine the significance. Statistical significance was defined as * for *p* < 0.05, ** for *p* < 0.01, *** for *p* < 0.001, and ns for no statistical significance (*p* > 0.05).

## 3 Results

We have previously shown that nucleolin and ribosomal protein S6, both involved in pre-rRNA transcription and ribosome assembly, are potential MVI binding partners in neurosecretory PC12 (Majewski et al., 2018). These observations prompted us to investigate the role of MVI in nucleolar and ribosomal functions.

### 3.1 Myosin VI is present within the nucleolus

We started the examination with identification of structural grounds for the presence of MVI in the nucleolus. For this, we performed an analysis using a nucleolar localization sequence detector, NoD, created on the basis of the data of 46 human-confirmed nucleolar localization signals (NoLS) (Scott et al., 2011). The analysis predicted the presence of one NoLS within the MVI heavy chain with the following sequence: FHRRLKVYHAWKSKNKKRNTETEQRAPKS (Figures 1A and B). This positively charged region with the pi value of 10.99 (calculated with a protein isoelectric point calculator, http://isoelectric.org) spans residues 1114 and 1142, situated within the MVI cargo-binding domain. Furthermore, it overlaps the bipartite nuclear localization signal (NLS) and contains both the RRL motif, involved in electrostatic interaction with MVI partners, and the positively charged region (WKSKNKKRN), involved in PIP_2_ binding (Tumbarello et al., 2013; Spudich et al., 2007).

PC12 cell fractionation and immunogold staining showed that MVI is present in the nucleolar fraction (Figures 1C–E). Furthermore, analysis of immunogold staining revealed that the presence of MVI within the nucleolus is increased upon cell stimulation with 56 mM KCl (Figures 1D and E). Quantification of MVI-associated gold particles revealed that MVI localizes mainly to the granular component (GC) and dense fibrillar component (DFC), but the majority was present within the GC. This distribution does not depend on stimulation, as the same fraction of MVI is visible in both sub-compartments regardless of stimulation (Figure 1E).

### 3.2 Interaction of MVI with protein markers of sub-nucleolar compartments

**FIGURE 2:**
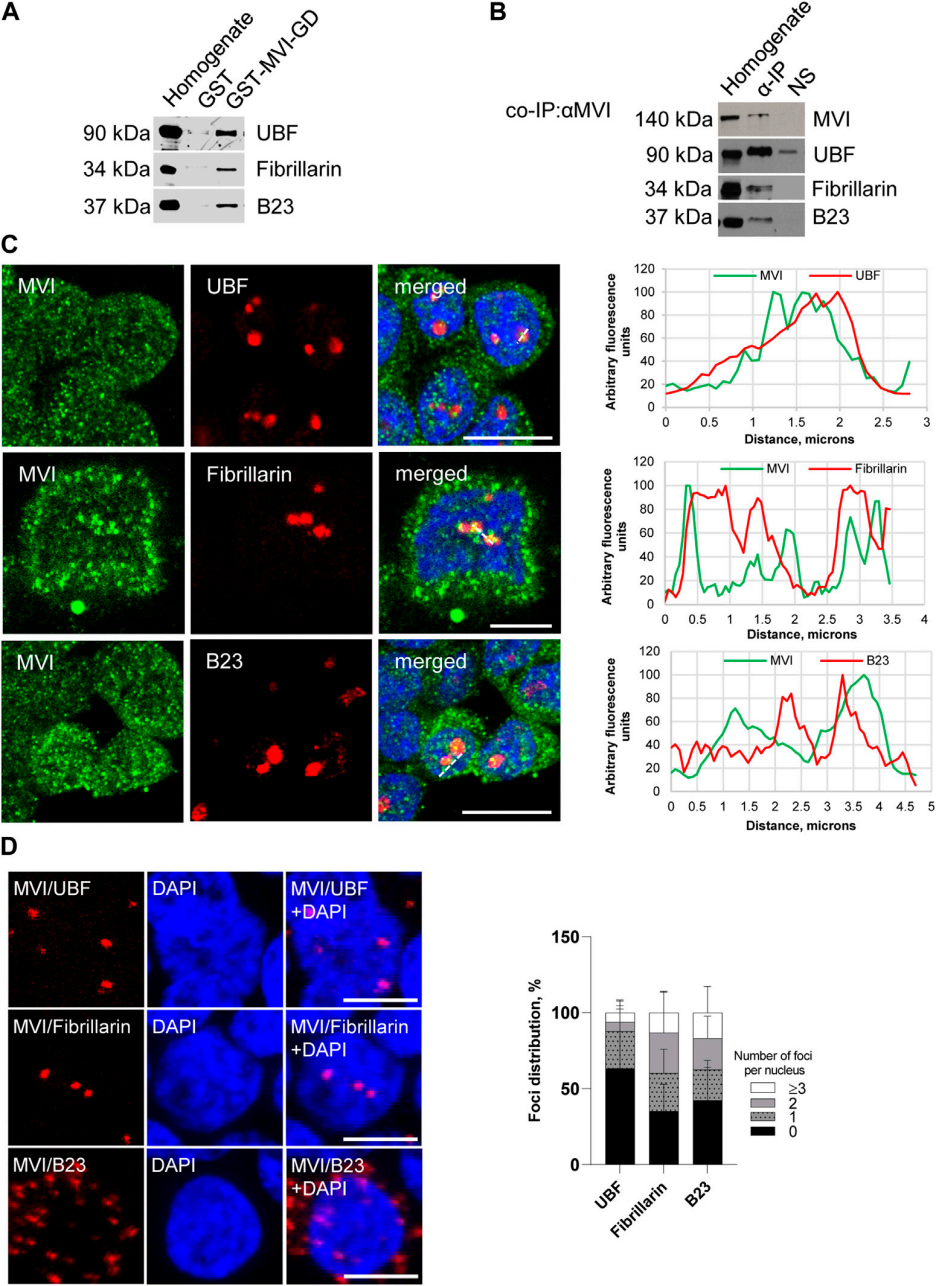
Assessment of the interaction of MVI with nucleolar proteins. **(A)** Immunoblot analysis of pull-down fractions. Homogenate, PC12 cell homogenate before loading onto Glutathione Sepharose; GST-MVI-GD, a fraction eluted from the resin with an attached MVI globular tail fused with GST, and GST, a fraction eluted from the GST-attached resin. The fractions were probed with antibodies against UBF, fibrillarin, and B23. **(B)** Co-immunoprecipitation of MVI/UBF, MVI/fibrillarin, and MVI/B23 with the anti-MVI antibody. Cell homogenates (homogenate), samples precipitated with anti-MVI antibody (α-IP) or with a non-immune serum (NS), were probed with anti-UBF, anti-fibrillarin, and anti-B23 antibodies, as marked on the figure. **(C)** Co-localization of MVI (in green) with UBF, fibrillarin, and B23 (in red). In blue, nuclei stained with DAPI. Images of the cell central sections (*z* = 0.3 μm) were obtained with a Zeiss LSM 780 confocal microscope. Bars, 10 μm. Left panels in C, fluorescence image profile and co-localization analyses; graphs represent fluorescence intensity profiles calculated on images obtained from samples co-immunostained for MVI and nucleolar proteins, as marked on the images with a dashed line. **(D)** PLA assay probing MVI/UBF, MVI/fibrillarin, and MVI/B23 interactions (in red) in PC12 cells. In blue, nuclei stained with DAPI. Images of the cell central sections (*z* = 0.3 μm) were obtained with a Zeiss LSM 780 confocal microscope. Bars, 10 μm. Graph in right, evaluation of PLA-positive foci, corresponding to the interaction of MVI with UBF, fibrillarin, and B23 per nucleus. 100% corresponds to all the examined nuclei (N = 64 for UBF, N = 59 for fibrillarin, and N = 87 for B23); detailed information is given in Supplementary Figure S2B).

To show whether MVI can interact with markers of nucleolar compartments, namely, UBF (upstream binding factor, marker of FC), fibrillarin (marker of DFC), and B23 (marker of GC), we performed a series of experiments using the immunoprecipitation (IP) as well as pull-down techniques, immunofluorescence staining, and proximity ligation assay (PLA) (Figure 2; Supplementary Figures S1, S2).

The pull-down assay with the MVI cargo-binding domain (GST-MVI-GD) as a bait, followed by immunoblotting, revealed that the selected marker proteins were present in the fractions precipitated with the MVI fragment and not with GST alone (Figure 2A). In addition, the analysis of the fractions co-IPed with the anti-MVI antibody (see Figure 2B) demonstrated the presence of the above-mentioned nucleolar proteins and MVI in the precipitates obtained upon incubation of cell lysates with the antibody but not with the control non-immune serum. It should be noted that the low yield of IP and pull-down assays for fibrillarin suggests that these interactions may be weak and/or transient.

Double immunostaining for MVI and the aforementioned nucleolar proteins showed their co-localization, further confirming their interaction with MVI (Figure 2C; Supplementary Figure S1).

To check whether these interactions also exist *in cellulo*, we employed the proximity ligation assay (PLA), designed for *in situ* detection of two proteins existing within close intracellular proximity (within the 20–40 nm range) (Soderberg et al., 2006). As shown in Figure 2D and Supplementary Figure S2A, red dots representing positive PLA signals indicative of the close proximity of two examined proteins were observed within the nucleus for UBF, fibrillarin, and B23. Thus, these data confirm the interaction of MVI with these proteins *in situ*. Positive signals in the perinuclear region were also detected for B23. Quantification of the number of PLA foci per nucleus revealed that the interaction does not take place in all the nuclei and varies between the examined proteins (Figure 2D; Supplementary Figure S2B). The highest fraction of “foci-positive” nuclei was observed for fibrillarin (∼65%), and the lowest for UBF (∼36%). As for B23, the fraction of “foci-positive” nuclei constitutes ∼57%, but in case of these interactions, we observed the highest fraction of the nuclei, which contained ≥3 foci (∼17%).

### 3.3 Effects of MVI depletion on the organization of the nucleolus

**FIGURE 3:**
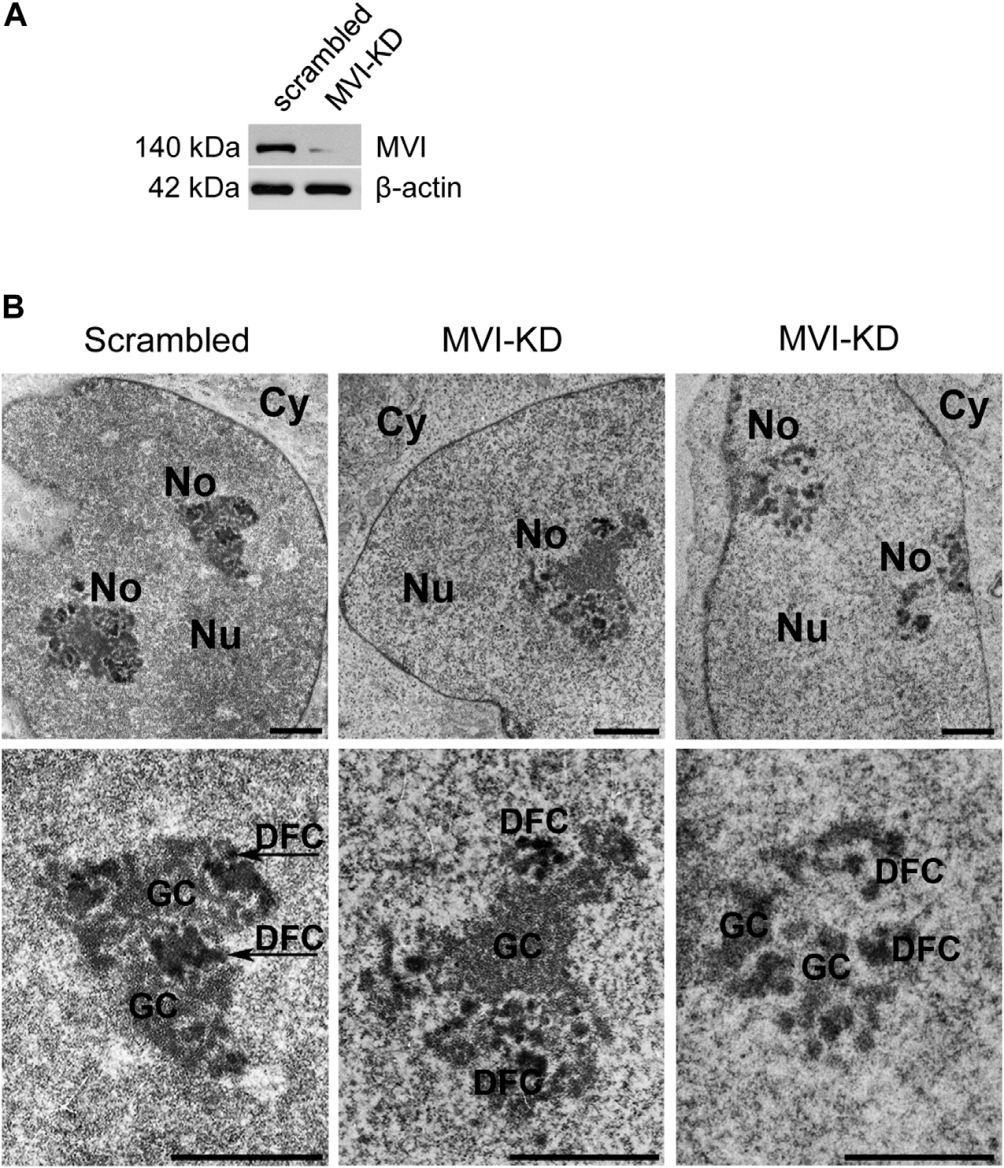
Effect of MVI depletion on morphology of nucleoli. **(A)** Immunoblot analysis of MVI in scrambled and MVI-KD PC12 cells. **(B)** Electron microscopy images of the nucleoli of scrambled and MVI-KD cells. Lower panels, ∼×2.5 magnification of the areas marked in the corresponding upper panels. Nu, nucleus; No, nucleolus; FC, fibrillar center; DFC, dense fibrillar component; GC, granular component. Bars, 1 µm.

Since MVI is known to be involved in the organization of cytoskeletal compartments, and in PC12 cells (Majewski et al., 2011), we tested whether and how depletion (by ∼90%) of this molecular motor (Figure 3A) affects the organization of the nucleolus. For this, we employed transmission electron microscopy, which showed different phenotypes, with more or less defective nucleoli in MVI-depleted cells (Figure 3B; Supplementary Figure S3). In the presented examples, the nucleoli are disorganized, with structural defects in both the DFC and GC subdomains. Furthermore, in some of the images, identification of these nucleolar compartments was not possible at all.

### 3.4 Effects of MVI depletion on localization of nucleolar proteins

**FIGURE 4:**
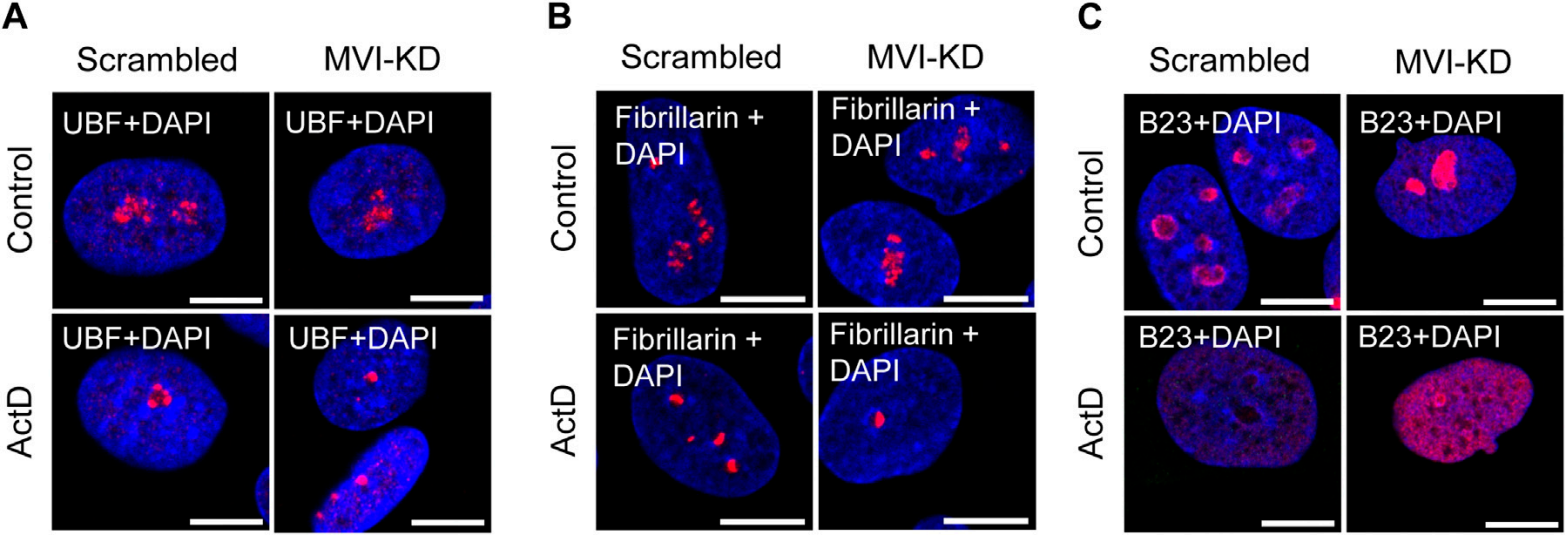
Effect of MVI depletion and actinomycin D on the localization of nucleolar proteins. **(A)** Localization of UBF (in red) in scrambled and MVI-KD cells after 3-h treatment with (ActD) and without (control) actinomycin D at the 0.05 μg/mL concentration. **(B)** Nucleolar localization of fibrillarin (in red) in scrambled and MVI-KD cells after 3-h treatment with (ActD) and without (control) actinomycin D at the 0.05 μg/mL concentration. **(C)** Redistribution of nucleolar protein B23 (in red) in scrambled and MVI-KD cells after 3-h treatment with (ActD) and without (control) actinomycin D at the 0.05 μg/mL concentration. In blue, nuclei stained with DAPI. Images of the cell central sections (*z* = 0.3 μm) were obtained with a Zeiss LSM 780 confocal microscope. Bars, 10 μm.

Since depletion of MVI causes disorganization of the nucleolus, we checked whether its presence is required for preserving the association of the examined nucleolar proteins with their locations. For this, we performed immunostaining for UBF, fibrillarin, and B23 in MVI-KD cells (Figure 4; Supplementary Figures S4–S6). Furthermore, the morphological changes observed in the nucleoli of MVI-KD cells suggest that MVI depletion may evoke conditions resembling nucleolar stress to some extent.

Therefore, we incubated MVI-KD cells with the Pol1 inhibitor, ActD, at the concentration of 0.05 μg/mL, known to inhibit the activity of this polymerase only (Reich et al., 1961). Since there is no information on the effect of ActD on the nucleoli of PC12 cells, we visualized the effects of this Pol1 inhibitor using transmission electron microscopy on the nucleoli of the examined cells (Supplementary Figure S7). As expected, the ActD treatment caused a collapse of the nucleoli with a segregation of its fibrillar and granular components (Yang et al., 2018; Lafita-Navarro and Conacci-Sorrell, 2023). In addition, we showed that ActD at this concentration does not affect the content of MVI in the cytoplasm and nuclear fractions of control cells (Supplementary Figure S8).

As presented in Figure 4A and Supplementary Figure S4, depletion of MVI did not affect the localization of UBF, as in both scrambled and MVI-KD cells, this protein was dispersed within the nucleolus. In control cells treated with ActD, UBF localized to the nucleolar caps shaped around the nucleolar remnants (Yang et al., 2018). Localization of this protein in the ActD-treated MVI-KD cells did not substantially differ from that in the control counterparts. A similar observation was made for fibrillarin (Figure 4B; Supplementary Figure S5). Thus, depletion of MVI in the presence or absence of ActD does not affect the localization of UBF and fibrillarin. Furthermore, MVI knockdown does not affect the overall level of these two proteins (Supplementary Figure S9).

However, the examination of localization of B23 revealed the evident difference between the two tested conditions (Figure 4C; Supplementary Figure S6). While in scrambled cells, this protein mostly localized to the nucleolar periphery, in MVI-KD cells, it was present within the entire nucleolus. Treatment of both cell types with ActD caused delocalization of B23 to the nucleoplasm, but in MVI-depleted cells, incubated with the inhibitor, the nucleoplasmic staining for B23 was more prominent, and the protein was still present at the edges of the nucleolar remnants (Figure 4C; Supplementary Figure S6). Immunoblotting analysis did not reveal statistically significant changes in the level of B23 in MVI-KD lysates (Supplementary Figure S9).

### 3.5 Examination of involvement of MVI in Pol1 activity

**FIGURE 5:**
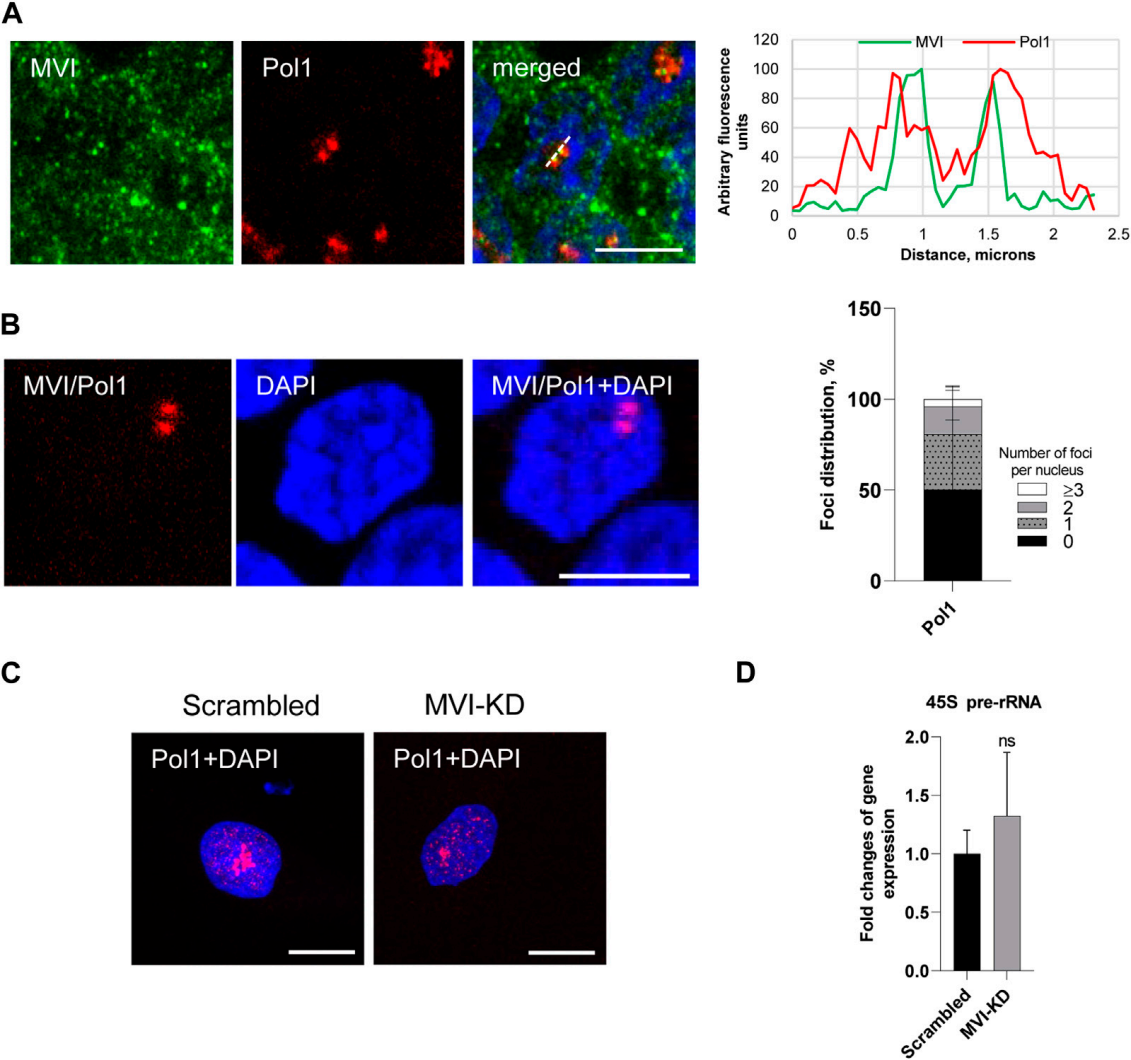
Effect of MVI depletion on Pol1 activity. **(A)** Co localization of MVI (in green) with Pol1 (in red). In blue, nuclei stained with DAPI. Right panel in B, fluorescence image profile and co localization profiles calculated on images obtained from samples co-immunostained for MVI and Pol1, as marked on the image with a dashed line. **(B)** PLA assay probing the MVI/Pol1 interaction (in red) in PC12 cells. In blue, nuclei stained with DAPI. Graph in right, evaluation of PLA-positive foci, corresponding to the interaction of MVI with Pol1 per nucleus. 100% corresponds to all the examined nuclei (N = 40, detailed information is given in Supplementary Figure S10. **(C)** Immunostaining for Pol1 (in red) in scrambled and MVI-KD cells. Nuclei were stained with DAPI (in blue). Images in B and C of the cell central sections (*z* = 0.3 μm) were obtained with a Zeiss LSM 780 confocal microscope. Bars, 10 μm. **(D)** Levels of 45S pre-rRNA in scrambled and MVI-KD cells were determined by quantitative real-time PCR and normalized against the levels of 18S rRNA. ns, not statistically significant. A quantitative analysis was based on assays performed in triplicates on four different cell cultures.

Next, we decided to test whether depletion of MVI could affect Pol1-based transcription (see Figure 5; Supplementary Figures S10 and S11).

As shown in Figures 5A and B and Supplementary Figure 10, Pol1 co-localizes with MVI and interacts with this motor protein *in situ*, as revealed by the immunofluorescence and PLA assays. Quantification of the number of PLA foci per nucleus revealed that the Pol1–MVI interaction takes place in about 50% nuclei (Figure 5B).

In addition, the depletion of MVI did not have an evident effect on Pol1 distribution (Figure 5C; Supplementary Figure 11).

To test whether MVI could be involved in Pol1-based transcription, we used the q-RT-PCR technique to examine whether the depletion of MVI affects the synthesis of 45S pre-rRNA transcript (Kalita et al., 2008). As shown in Figure 5D, there is no significant change in the amount of the transcript in MVI-KD cells with respect to control cells, suggesting that MVI does not play a significant role in Pol1 activity.

### 3.6 Involvement of MVI in ribosome organization

Our previous results indicate that in PC12 cells, MVI not only localizes to the endoplasmic reticulum (ER) membranes but also interacts with ribosomal protein S6 (Majewski et al., 2018), which suggests that this molecular motor might be involved in the ribosome and/or ER organization. Furthermore, our current observation that MVI is important for the maintenance of the nucleolar organization makes this suggestion plausible.

**FIGURE 6:**
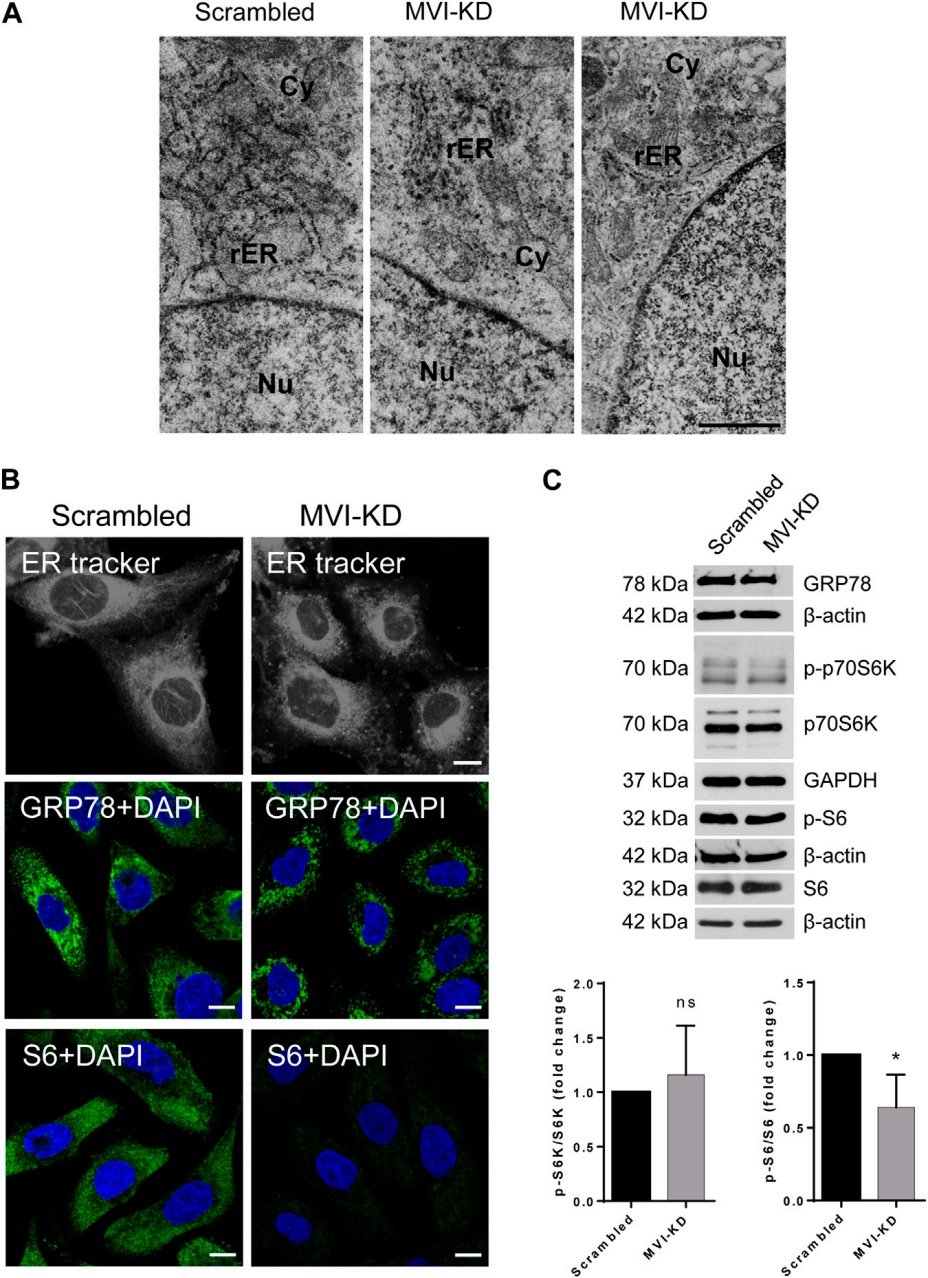
Effect of MVI depletion on endoplasmic reticulum organization (ER). **(A)** Electron microscopy visualization of the ER of scrambled and MVI-KD cells. Bars, 2 µm. **(B)** Visualization of the ER with fluorescent microscopy. Upper panels, the ER in scrambled and MVI-KD cell lines visualized by staining with the ER-specific dye, ER Tracker™ Blue/White DPX. Middle panels, immunostaining for GRP78 in scrambled and MVI-KD cells. GRP78 was visualized with anti-GRP78 antibody (in green) and nuclei with DAPI (in blue). Bottom panels, immunostaining for S6 in scrambled and MVI-KD cells. S6 was visualized with the anti-S6 antibody (in green) and nuclei with DAPI (in blue). Images in B of the central cell section (z = 0.3 µm) were obtained with a Zeiss LSM 780 confocal microscope. Bars, 10 μm. **(C)** Immunoblotting analysis of GRP78, p-S6, and S6 as well as of p-p70S6K and p70S6K in scrambled and MVI-KD PC12 cells. β-actin and GAPDH served as protein loading controls. Graphs, ratio of p-p70S6K to p70S6K levels (on the left) and p-S6 to S6 levels (on the right). 1, the levels of the examined proteins in scrambled cells. *, *p* < 0.05; ns, not statistically significant.

The analysis of the TEM images revealed that in MVI-KD cells, the ER membranes are not only inflated but also less decorated with the ribosomes with respect to control scrambled cells (Figure 6A). In addition, live staining for the ER membranes with the ER Tracker showed profound changes in the ER organization in MVI-KD cells (Figure 6B, upper panels).

Immunostaining for GRP78, a key protein involved in protein folding and quality control in the ER (Kleizen and Braakman 2004), confirmed the disorganization/fragmentation of this membranous compartment in MVI-KD cells (Figure 6B, middle panels). Immunostaining for S6 (Figure 6B, bottom panels) revealed a very weak signal in MVI-KD cells. This is in contrast to our observation that the overall level of S6, similarly to GRP78, was not affected by MVI depletion (Figure 6C). This could be explained in terms that depletion of MVI might impair the availability of an S6 epitope *in cellulo*. We also examined the level of p70S6K (and its active, phosphorylated form, p-p70S6K), a kinase involved in regulation of S6 activity. As presented in Figure 6C, the ratio of p-p70S6K to p70S6K level is not affected by MVI depletion, but the ratio of p-S6 to S6 is decreased, indicating that the activity of S6 is associated with MVI.

## 4 Discussion

Our study demonstrates for the first time that in neurosecretory PC12 cells, MVI interacts with several nucleolar proteins and seems to play a role in nucleolus and ribosome maintenance, though it seems not to be crucial for Pol1-dependent transcription.

MVI translocates to the nucleus due to the presence of several nuclear localization signals (NLS) within its heavy chain (Majewski et al., 2018; Vreugde et al., 2006, Hari-Gupta el al., 2022). Here, we show that MVI also contains one putative nucleolar localization signal (NoLS), situated within the MVI cargo-binding domain, overlapping the bipartite NLS and containing motifs involved in the interaction with MVI partners and phospholipids (Figure 1A) (Majewski et al., 2018; Spudich et al., 2007; Tumbarello et al., 2013). It is plausible that this region may be involved in the MVI presence in the nucleolus, though the specificity of this putative NoLS is not clear yet. It is noteworthy that the presence of two functional NoLS regions was confirmed within myosin IC heavy chain (also termed as NMIC) that are necessary for its nucleolar presence, specifically for isoform B of NMIC (Schwab et al., 2013). Of note, there are three NMIC posttranslational forms A, B, and C, and two of them, B and C, localize to the nucleus (Ihnatovych et al., 2012). One NoLS is located within the N-terminal positively charged peptide specific for this NMIC isoform, and the second is present upstream of the neck region within the head domain (Schwab et al., 2013). The authors postulate that this is a mechanistic explanation for the observed functional differences between the NMIC isoforms. Interestingly, a different mechanism is behind the nuclear/nucleolar translocation of myosin VA (MVA), one of the three myosin isoforms reported to be located in the nucleolus. It has been shown in neurons that while phosphorylation of Ser1650 of MVB by CaMKII is crucial for its presence in the nucleus (where it localizes within the speckles), inhibition of transcription causes its redistribution to the nucleolus (Pranchevicius et al., 2008). Though there is no report on how myosin VB (MVB) translocates to the nucleus/nucleolus, it is plausible that the same mechanism is employed as the region corresponding to the amino acid sequence of MVB reveals a high degree of similarity to the MVA isoform (Rodriquez and Cheney 2002).

Localization of MVI to the nucleolar subdomains as well as its newly revealed interaction with proteins specific to these regions, UBF, fibrillarin, and B23 suggest the involvement of MVI in ribosome biogenesis taking place in this nuclear compartment. However, based on these analyses, we cannot state whether or not MVI directly interacts with these nucleolar proteins or is simply a part of a complex containing one or all of them. In addition, our earlier observation on the interaction of MVI with nucleolin seems to strengthen this suggestion (Majewski et al., 2018). Furthermore, a shift in the localization of B23 in MVI-depleted cells, especially under nucleolar stress conditions, additionally confirms our suggestion regarding the important role of this molecular motor in nucleolar functions. In addition, data showing that depletion of MVI affects the nucleolar structure and ER organization, particularly the decrease in ribosome decoration, further confirm this suggestion. Notably, disorganization of the ER was also observed in MVI-depleted myoblasts (Karolczak et al., 2015).

However, despite co-localization of MVI with Pol1, its depletion does not seem to affect this polymerase activity. So far, two unconventional myosins, MVB and NMIC, have been found to be associated with Pol1 complexes (Lindsay and McCaffrey 2009; Philimonenko et al., 2004). While for MVB, the mechanisms of its involvement in nucleolar transcription have not yet been elucidated, much more is known about the involvement of NMIC. It has been shown that it plays a role in both the maturation and export of competent pre-ribosomal subunits to the nuclear pore complex (Obrldik et al., 2010). Furthermore, the presence of NMIC also facilitates the modification of histone H3 at Lys9 (H3AcK9) that is required to activate rRNA gene transcription and cell cycle progression (Sarshad et al., 2013). In line with this is our recent observation that in the nuclei of PC12 cells MVI co-localizes with the same modification of histone 3 (Majewski et al., 2018). In addition, in our cell model, MVI interacts with several proteins such as hnRNPs and SC35 (Majewski et al., 2018) as well as the above-mentioned nucleolin, UBF, fibrillarin, and B23, all involved in maturation and processing of the products of the Pol1 and/or Pol2 transcription. We hypothesize that interactions with these newly identified nucleolar partners could be responsible for engagement of MVI in processes taking place in the nucleolus.

Thus, the question arises about the mechanism(s) behind the observed effects of MVI depletion on the organization of the nucleolus and ribosome-containing ER membranes. Based on our and other groups’ data, it is known that MVI interacts with numerous partners specific not only for the cell/tissue type but also for the MVI isoform present in a given cell/cellular compartment (Majewski et al., 2018; Wollscheid et al., 2016; Tumbarello et al., 2013; Dos Santos et al., 2022). Recently, we showed that in PC12 cells, the dominant one is the isoform without the large insert, which is able to shuttle between the cytoplasm and the nucleus in a stimulation-dependent manner (Majewski et al., 2018). The data presented herein, showing that the number of MVI-positive particles is elevated upon stimulation, indicate that upon cell stimulation, MVI can also translocate to the nucleolus (either from the nucleoplasm and/or directly from the cytoplasm), where it can interact with nucleolar proteins and, thus, be involved in nucleolar processes. Its presence is important for the maintenance of the nucleolus morphology, indicating that it may act here as the crosslinker stabilizing the structure *via* the interaction between the nuclear/nucleolar actin pool(s) and nucleolar proteins (Ulferts et al., 2021). Interestingly, similar effects on nucleolar morphology have been demonstrated for fascin, an actin filament-bundling protein primarily known for its involvement in the promotion of cell migration (Lamb and Tootle, 2020). The other possibility is that depletion of MVI impairs the import of the nucleolar components from the cytoplasm as MVI can act as a transporting motor as well (Cook et al., 2020). In addition, the lack of MVI may affect the export of the products of the nucleolar processes, and this could be responsible for the observed disorganization of the ER membranes and the reduced ribosome load. Involvement of MVI in ribosome biogenesis/assembly could also result from its interaction with ribosomal protein S6 and, in particular, with its active phosphorylated form (p-S6), which is a downstream effector of mTOR kinase (Majewski et al., 2018; Meyuhas 2015). Of note, MVI–S6 interactions have been also shown for skeletal muscle and myogenic cells [Lehka et al., unpublished]. It is noteworthy that an ATP-dependent spatial association of S6-containing small ribosomal subunits (SSUs) with NMIC and actin within the nuclear compartments and at the nuclear pores has been shown in HeLa cells (Cisterna et al., 2006; Cisterna et al., 2009). The authors’ suggestion that NMIC is involved in the actin-dependent export of SSUs to the cytoplasm supports our hypothesis that MVI could be engaged in transporting ribosome components to and from the nucleus/nucleolus. The MVI–S6 interaction has not yet been characterized at the molecular level, but our staining for S6 in MVI-depleted cells seems to indicate that loss of MVI could affect the conformation of this 29-kDa protein of the 40S ribosomal subunit, as it was revealed upon RNA binding, indicating a direct interaction (Draper and Reynaldo 1999).

In summary, our study indicates for the first time that MVI is involved in nucleolar processes and ribosome biogenesis through its interaction(s) with the proteins involved in nucleolar and ribosome functions. However, further studies are needed to characterize these interactions at the molecular level.

## Data Availability

The original contributions presented in the study are included in the article/Supplementary Material; further inquiries can be directed to the corresponding author.
