# Supplementary material for: Myosin VI in the nucleolus of neurosecretory PC12 cells: its involvement in the maintenance of nucleolar structure and ribosome organization
Source: Front Physiol. 2024 May 7;15:1368416. doi: 10.3389/fphys.2024.1368416 (PMC11106421; doi:10.3389/fphys.2024.1368416)
Supplement: Supplementary file 1 [file DataSheet1.pdf]

Supplementary Material

Myosin VI in the nucleolus

Jolanta Nowak, Robert Lenartowski, Katarzyna Kalita, Lilya Lehka, Olena Karatsai, Marta Lenartowska and Maria Jolanta Rędowicz*

* Correspondence: Maria Jolanta Rędowicz: j.redowicz@nencki.edu.pl

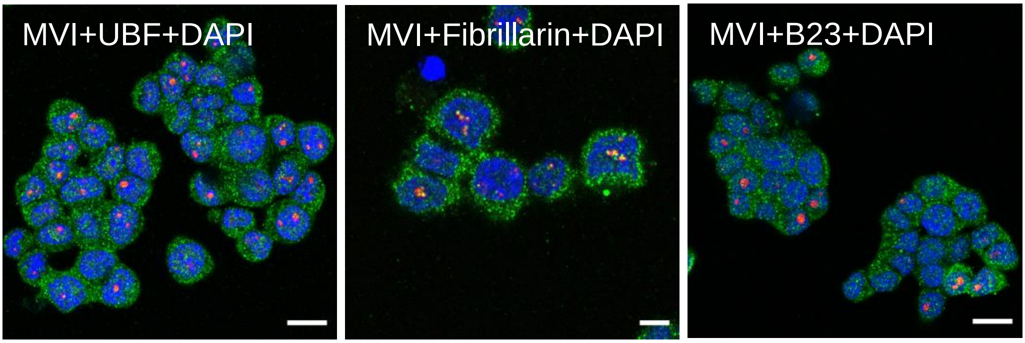

Supplementary Figure 1. Co-localization of MVI (in green) with UBF, fibrillarin and B23 (in red). In blue, nuclei stained with DAPI. Bars, 10 μm

(A)

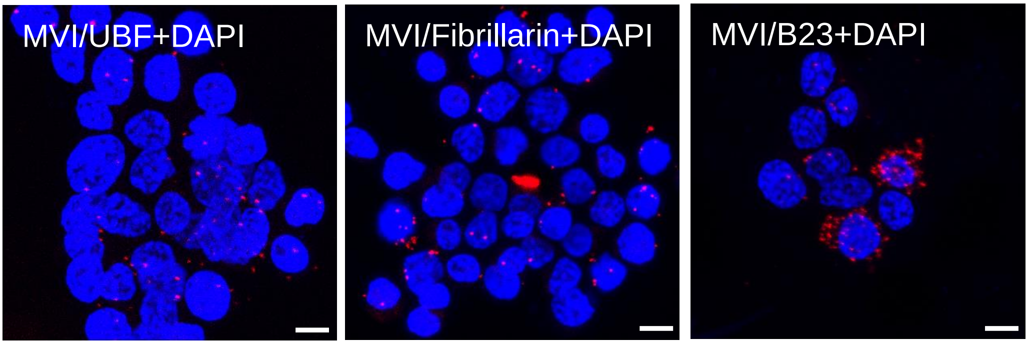

(B)

	Number of foci per nucleus			
	0	1	2	≥3
	Foci distribution, %			
UBF	63.3±39.0	24.3±20.8	6.2±10.1	6.2±7.7
Fibrillarin	35.0±18.0	25.2±15.8	26.7±13.2	13.2±14.1
B23	42.2±21.8	20.2±6.1	20.7±14.5	16.9±17.3

Supplementary Figure 2. (A) PLA assay probing MVI/UBF, MVI/fibrillarin and MVI/B23 *in cellulo* interactions (in red) in PC12 cells. In blue, nuclei stained with DAPI. Bars, 10 μm. (**B)** Quantification of the number of PLA foci per nucleus were counted manually; n=64 nuclei (for UBF), n=59 nuclei (for fibrillarin), n= 87 nuclei (for B23).

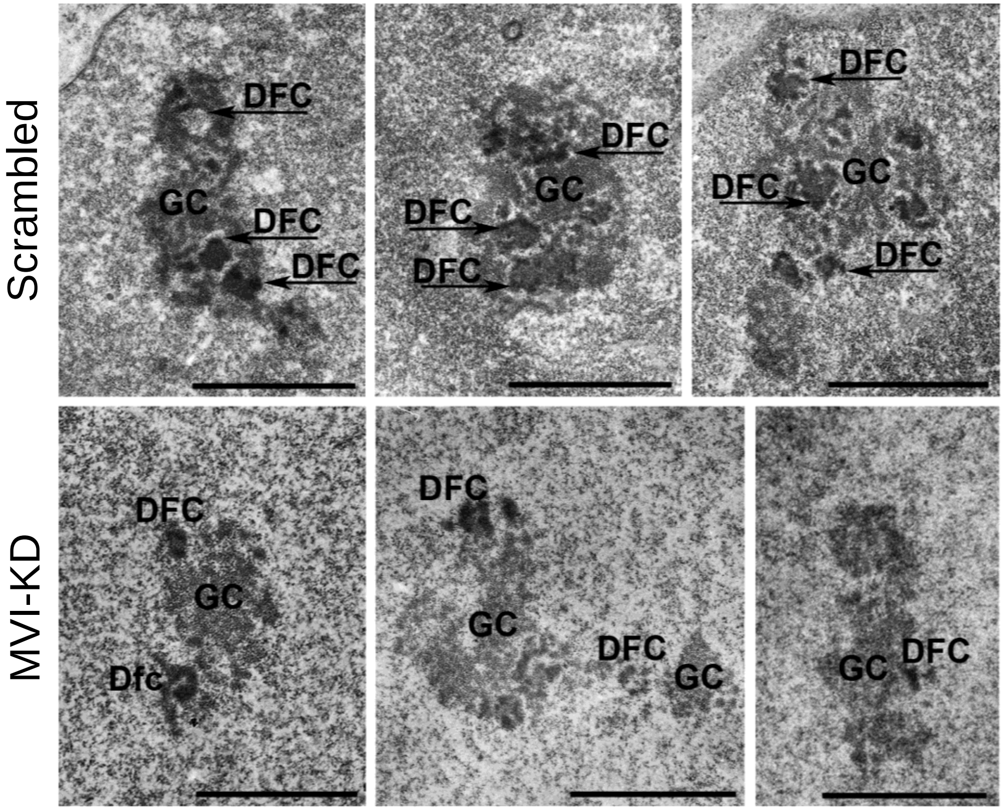

Supplementary Figure 3. Electron microscopy images of nucleoli of scrambled and MVI-KD cells. DFC, dense fibrillar component, GC, granular component. Bars, 2 μm.

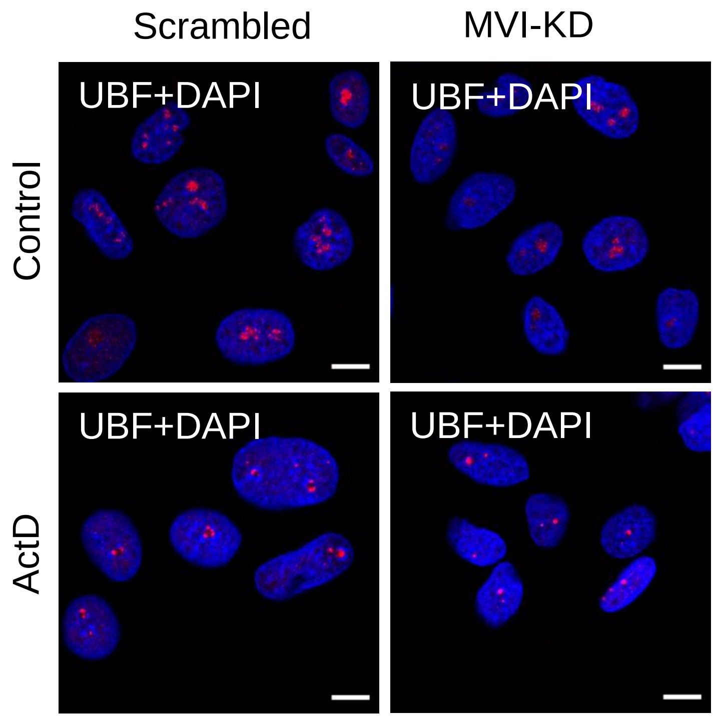

Supplementary Figure 4. Nucleolar localization of UBF in scrambled and MVI-KD cells after 3-h treatment with (ActD) and without (control) 0.05 µg/mL of actinomycin D. The formation of UBF-positive nucleolar caps was found after treatment with ActD. In blue, nuclei stained with DAPI. Bars, 10 μm.

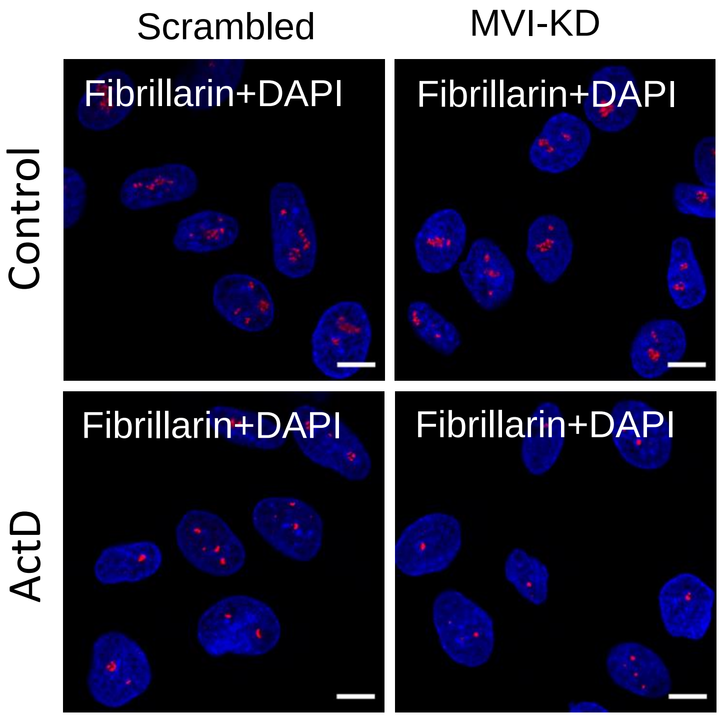

Supplementary Figure 5. Nucleolar localization of fibrillarin in scrambled and MVI-KD cells after 3-h treatment with (ActD) and without (control) 0.05 µg/mL of actinomycin D. The formation of fibrillarin-positive nucleolar caps were found after treatment with ActD. In blue, nuclei stained with DAPI. Bars, 10 μm.

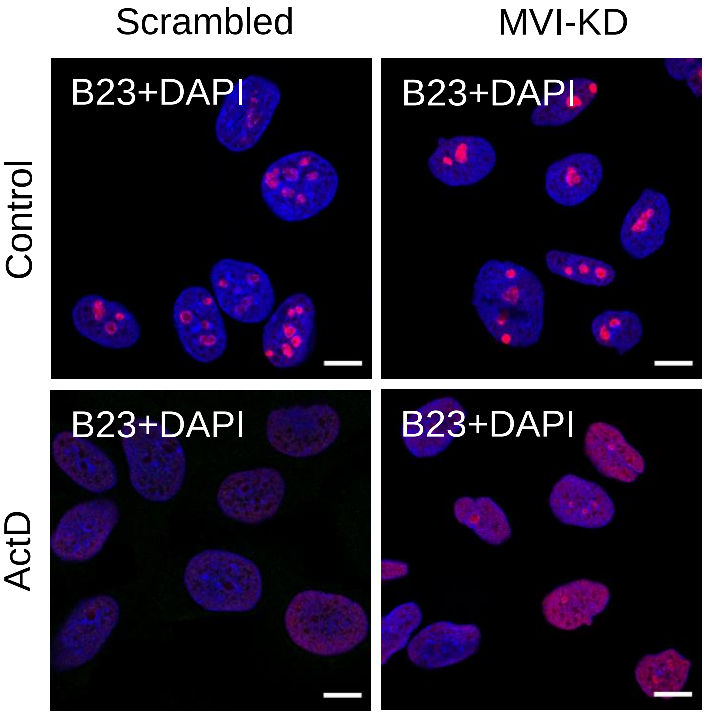

Supplementary Figure 6. Redistribution of nucleolar protein B23 in scrambled and MVI-KD cells after 3-h treatment with (ActD) and without (control) 0.05 µg/mL of actinomycin D. Upon ActD treatment B23 translocated from the nucleoli to the nucleoplasm in a diffusive pattern, however a fraction of B23 stayed at the nucleolar cups in MVI-KD cells. In blue, nuclei stained with DAPI. Bars, 10 μm.

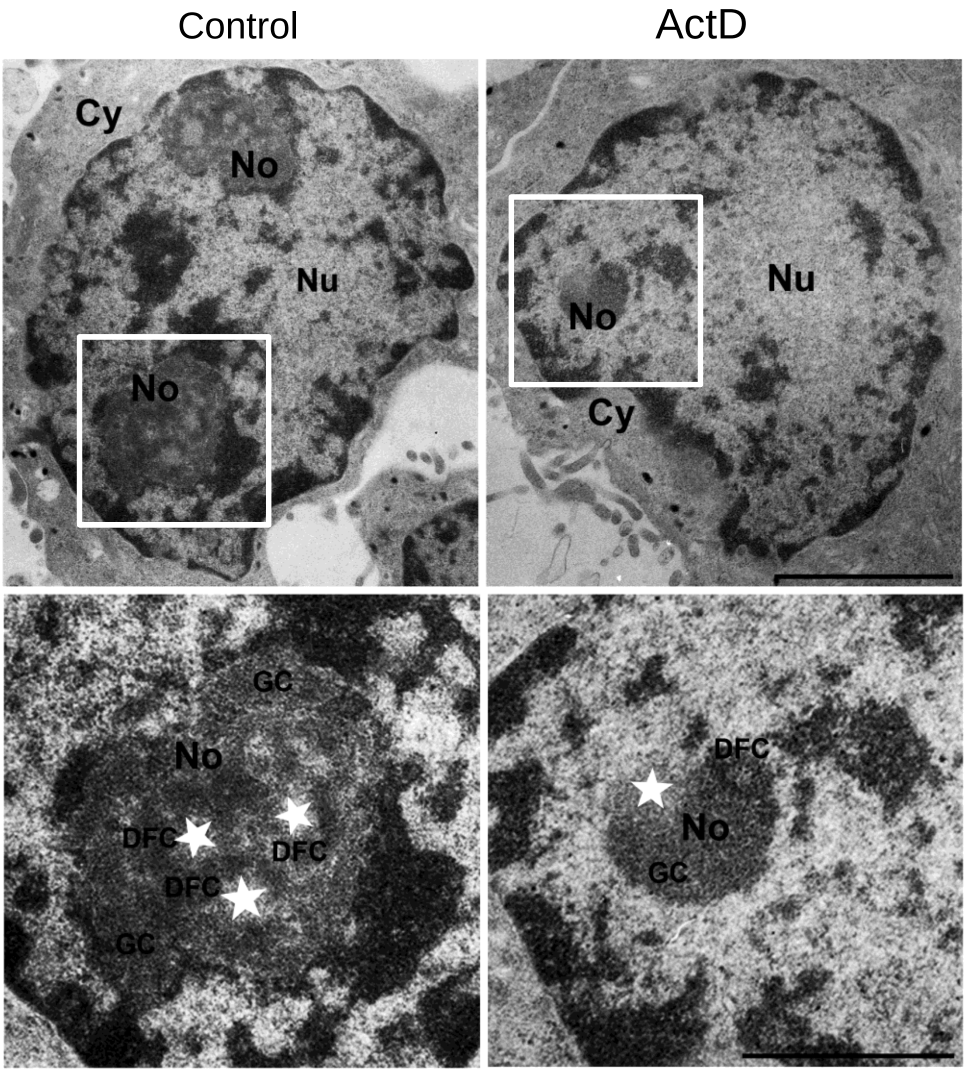

Supplementary Figure 7. Electron micrographs of nucleoli of PC12 cells with (ActD) and without 3-h treatment (control) with 0.05 μg/mL actinomycin D. Left panels, in control conditions nucleolus with several fibrillar centers (asterisks) surrounded by dense fibrillar component (DFC) and globular component (GC). Right panels, reorganisation of nucleolar components induced by the inhibition of rRNA synthesis by ActD with remnants of density fibrillar centers (asterisk) segregated at the nucleolar periphery. Lower panels, ~2.5 magnification of areas marked in upper panels, Bars, 2 μm (upper panels) and 1 μm (lower panels). Cy-cytoplasm, No-nucleolus, Nu-nucleus.

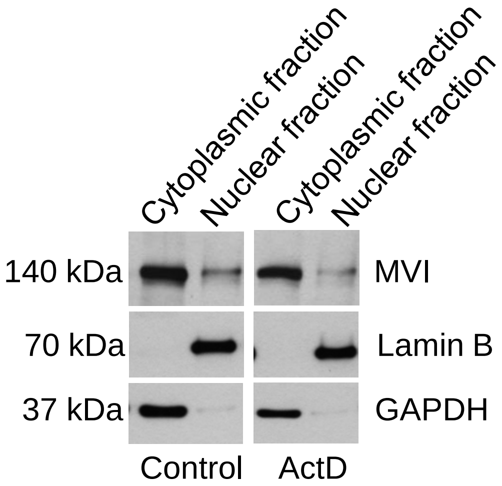

Supplementary Figure 8. Detection of MVI in nuclear and cytoplasmic fraction of PC12 cells with (ActD) and without (control) treatment with 0.05 µg/mL actinomycin D. Lamin B, nuclear protein marker was detected only in the nuclear fraction; GAPDH, cytoplasmic protein marker was detected only in the cytoplasmic fraction.

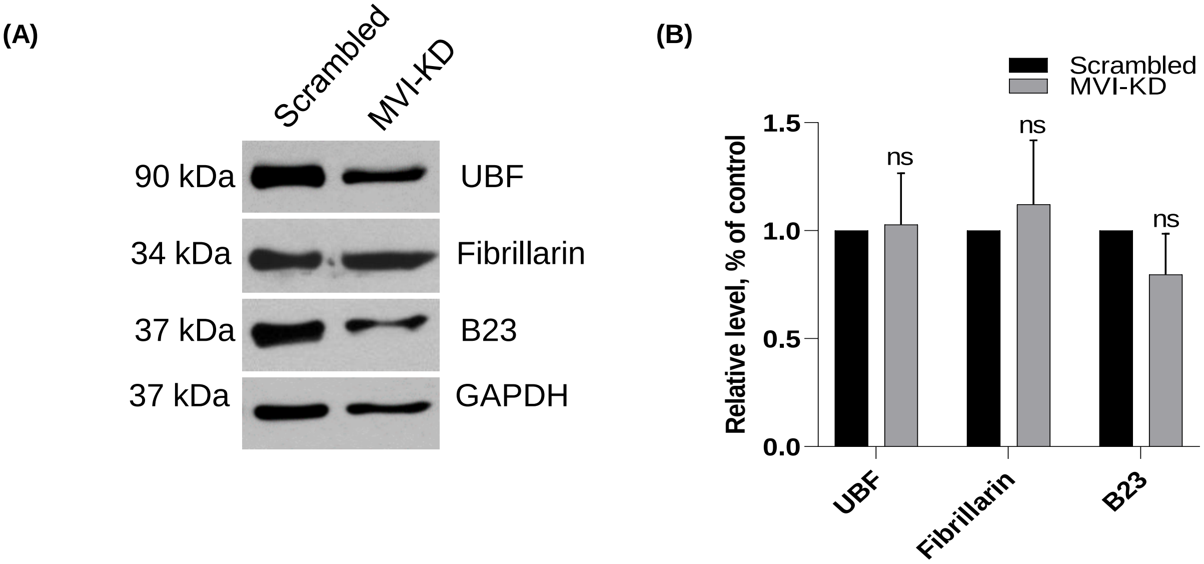

Supplementary Figure 9. (A) immunoblot analysis of UBF, fibrillarin and B23 in scrambled and MVI-KD PC12 cells. GAPDH served as an internal protein loading control. **(B)** Densitometric analysis of immunoblots probed for UBF, fibrillarin and B23 in scrambled and MVI-KD PC12 cells. ns, statistically not relevant. Analysis was based on immunoblots of cell lysates derived from three different cell cultures. ns, not statitically significant.

(A) (B)

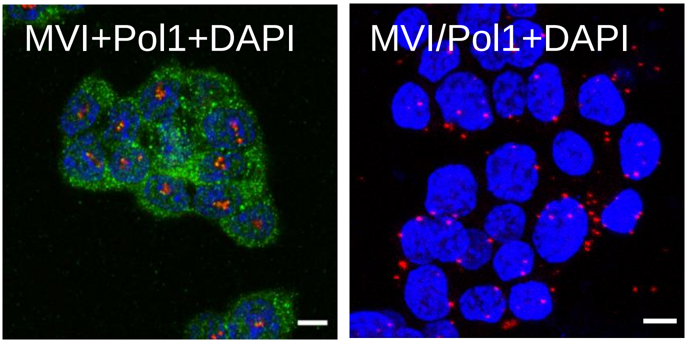

(C)

	Number of foci per nucleus			
	0	1	2	≥3
	Foci distribution, %			
Pol1	50.3±38.2	30.4±26.7	15.4±8.9	3.9±6.8

Supplementary Figure 10. Assessment of MVI-Pol1 interaction. (**A)** Co-localization of MVI (in green) with Pol1 (in red). In blue, nuclei stained with DAPI. Bars: 10 µm. (**B)** PLA assay probing the MVI/Pol1 interaction (in red) in PC12 cells. In blue, nuclei stained with DAPI. Bars, 10 µm. (**C)** the number of PLA foci per nucleus were counted manually; n=40 nuclei.

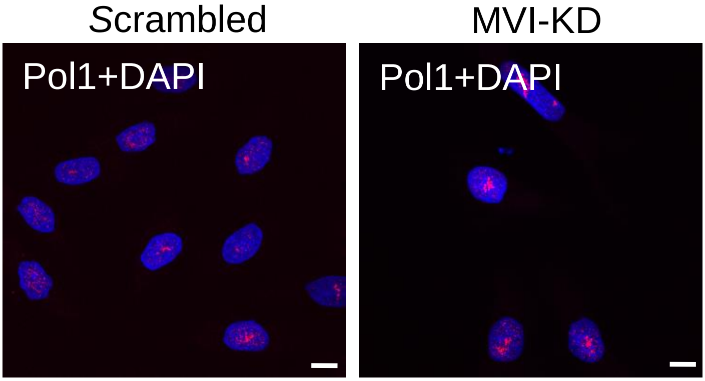

Supplementary Figure 11. Immunostaining for Pol1 in scrambled and MVI-KD cells. Pol1 was visualized with anti-Pol1 antibody (in red), nuclei with DAPI (in blue). Bars, 10 µm.
